# Continuity of Constitutional Government during a Pandemic: Considering the Concept in Canada's Emergency Management Act

**DOI:** 10.1017/S0008423920000293

**Published:** 2020-04-14

**Authors:** Andrew McDougall

**Affiliations:** Department of Political Science, University of Toronto, 1265 Military Trail, Highland Hall 548, Scarborough, ON, M1C 1A4

## Abstract

This research note examines the undefined meaning of the government's obligations to ensure “continuity of constitutional government” (CCG) as provided for in section 4(l) of the Emergency Management Act, S.C. 2007, c. 15 (Canada, 2007). Specifically, that section gives the minister of public safety and emergency preparedness the responsibility for “establishing the necessary arrangements for the continuity of constitutional government in the event of an emergency,” but the term is itself undefined. The article will canvass the origin of the term and its relationship to other so-called continuity of government (COG) concepts, along with some legal written opinion on what the term might in practice mean, should the minister ever be charged with discharging this responsibility. It will conclude with some final thoughts on the relevance of the CCG concept to the current pandemic. Given that the COVID-19 virus has infected Sophie Grégoire Trudeau, the prime minister's wife, forced a drastically reduced and possibly “virtual” federal Parliament, placed the British prime minister in intensive care and led to the self-isolation of many world leaders, the topic is relevant to Canada in 2020.

## Introduction

This research note examines the undefined meaning of the government's obligations to ensure “continuity of constitutional government” (CCG) as provided for in section 4(l) of the Emergency Management Act, S.C. 2007, c. 15 (Canada, 2007). Specifically, that section gives the minister of public safety and emergency preparedness the responsibility for “establishing the necessary arrangements for the continuity of constitutional government in the event of an emergency,” but the term is itself undefined. The article will canvass the origin of the term and its relationship to other so-called continuity of government (COG) concepts, along with some legal written opinion on what the term might in practice mean, should the minister ever be charged with discharging this responsibility. It will conclude with some final thoughts on the relevance of the CCG concept to the current pandemic. Given that the COVID-19 virus has infected Sophie Grégoire Trudeau, the prime minister's wife, forced a drastically reduced and possibly “virtual” federal Parliament, placed the British prime minister in intensive care and led to the self-isolation of many world leaders, the topic is relevant to Canada in 2020.

## Background

There are essentially three types of emergency plans that governments have, and the terminology distinguishing them is not always clear. *Emergency management* or *emergency governance* is what the country faces now, with the COVID-19 pandemic. All the major political and military leadership is intact, and these leaders are making use of the authorities they have been given through parliamentary action to manage the crisis (see, for example, Federal/Provincial/Territorial Ministers, 2017; Cunningham, 2013: 10). *Continuity of government*—sometimes *continuity of operations* (COOP)—is a different term, one that refers primarily to planning done in anticipation of a catastrophic event that decapitates the executive but is less concerned with the other branches or the administrative state (Graff, 2017: xix). In the event of the death or incapacity of the prime minister, it would fall to the governor general (or the lieutenant-governor, in the case of the provinces) to select a new head of the government in consultation with party leaders—namely, the person best positioned to maintain the confidence of Parliament or Legislature before a longer-term arrangement is made. In Canada, since sovereignty is always vested in the Crown, from a technical perspective, it is more important to ensure that there is no doubt who is on the throne than who is the head of government.

*Continuity of constitutional government* is a third and even bigger idea, however, because it contemplates the survival not just of the executive but of all the necessary actors and institutions in the Constitution to ensure longer-term continuity. In the United States, the idea is known as the *enduring constitutional government*, which is functionally the same thing and is aimed at preserving the “spirit” of the Constitution after a disaster (Graff, 2017: 375). In Canada, this would presumably include not just the prime minister but his or her cabinet, along with some minimum complement needed for the legislative and judicial branches to continue functioning and probably provision for other levels of government and constitutional actors.

There is relatively little, if any, theoretical treatment of CCG plans, most of which originated during the Cold War. Much of the work that has been done since then is American and followed the attacks of September 11, 2001, as part of renewed interest in government powers during emergencies or justifiable limitations on rights in crisis contexts (for a Canadian example, see Roach, 2008).^1^ In Canada, a notable effort to bring clarity to the CCG concept was recently presented to the Canadian Forces College by Lieutenant-Colonel J. W. Cunningham of the Canadian Forces in a master's thesis in 2013. He argues that all such plans must include five elements: *prevention* (of the disaster in the first place), *protection* (of key individuals and institutions), *succession* (in the event of the death of a key individual), *relocation* (in the event the venue of government is rendered unusable) and *reconstitution* (how to reassemble the Constitution in an effective and representative matter) (Cunningham, 2013: 5–6). Still, since the concept is undefined in legislation, it is unclear if Canada's plans in this regard, to the extent they exist, would pass muster.

The absence of clarity may, to a degree, be intentional. In the United States, the plans for enduring constitutional government have been made, but they are mostly classified (Graff, 2017: 375). This is not surprising, given that many of them contemplate a world at such an abstract level of disaster that it is doubtful there would be anyone left to implement them. Thus the aim of these plans is to “preserve the spirit of the constitution, not the letter of it” (Graff, 2017: 375). There are understandable reasons why the government may wish to avoid scrutiny of such plans, for both defence and political reasons. Nevertheless, they have an important place in today's context, where there is a significant natural disaster overtaking the planet and disrupting normal political channels, and it is worth considering Canada's definition of CCG.

## Welcome to the Bunker: The Ultimate Exercise in Elite Accommodation

Even though the concept remains undefined, some publicly available material has been prepared by government officials to consider what CCG might mean. A 2012 briefing package for the deputy minister of public safety unofficially defined CCG as the “principle of establishing defined plans and procedures that allow the three branches of the constitutional Government of Canada to continue to conduct essential operations in case of an emergency or catastrophic event in the National Capital Region” (cited in Cunningham, 2013: 12). But that clearly leaves many questions unanswered. Some further clarity might be found in a legal opinion commissioned to explore in more detail what the principle required. One interesting effort was made in 1990, when Emergency Preparedness Canada commissioned a study to answer what CCG would mean, from a legal point of view, in terms of how many people had to be saved to ensure constitutional continuity. Specifically, the authors, V. Seymour Wilson and Daniel Osabu-Kle, and two subcontractors were charged with answering several questions, including the meaning of the term *constitutional government*, as defined in the Emergency Management Act, and the minimum number of positions in each of the branches of government whose survival must be protected in order to ensure the continuity of legitimate constitutional government. The authors argued, with a rather macabre take, that one could take a “narrow” or “broad” view of who might need to be included to ensure continuity of constitutional government.

Narrowly speaking, the authors identified roughly 150 people who would be needed to maintain constitutional government in Canada; these are listed in Table 1 (with some amendments).^2^ In addition to those listed, you would need to add some “administrative capacity,” bringing it to “at least 100 people” (Wilson and Osabu-Kle, 1990: 7).

**Table 1:** Continuity of Constitutional Government: The Narrow View

Official	Number
The governor general and her successor, the chief justice	2
The Cabinet quorum of ministers	4
The lieutenant-governors of the ten provinces	10
A quorum of Cabinet in each province	~40
Additional administrative capacity	~40–50

Table adapted from Wilson and Osabu-Kle, 1990.

Clearly, the narrow view is more of a COG, rather than a CCG, outline of who is needed to continue government operations. Indeed, under the narrow view, the authors suggest it is only what Emergency Preparedness Canada was legally required to do, coordinating with other institutions and provinces that would otherwise be providing for their needs on their own. The study was also expressly comparative and examined similar legislation in several countries, noting that many plans differ on how much of the other branches needed to be saved by federal action. Most countries provide for the military and executive, along with the basic administration. They note that while the United States had plans for the judiciary, “other jurisdictions seem to omit this branch” (Wilson and Osabu-Kle, 1990: 6). By contrast, most countries made “ample provision for the preservation of the legislative branch” (6–7), perhaps reflecting the priorities of those who drafted the legislation.

A more generous, substantive accounting, and one more in line with CCG principles, provided that there be at least 300 persons or so, reproduced in Table 2, again with some amendments:

**Table 2:** Continuity of Constitutional Government: The Broad View

Official	Number
The governor general	1
The Cabinet	4
Members of the House of Commons	20
Members of the Senate	15
Speakers of both houses	2
Supreme Court	5
Federal Court (Trial Division)	1
Federal Court of Appeal	3
The clerk of the Privy Council and the secretary to Cabinet	1
The secretary to the Cabinet for Federal-Provincial Relations (or similar official)	1
The Privy Council's Committee of Senior Officials (COSO)	~9
The top two senior officials in the prime minister's office	2
The chief of the defence staff, and three top military commanders of the army, navy and air force	4
Senior officials of Emergency Preparedness Canada	~4
Additional administrative capacity	~200
Provincial lieutenant-governors	10
Provincial premiers	10
Advisers	~100

Table adapted from Wilson and Osabu-Kle, 1990.

What of the provinces? Again, the position of the report was that the act could be read to require only that the federal government cooperate with the provinces and that the provinces are each responsible for their own planning, which they “zealously” guard (Wilson and Osabu-Kle, 1990: 9). Still, the authors point out that under the broad view, the federal level should be expected to provide refuge to officials if requested by the provinces. One could further argue it would be a constitutional imperative to do so. It is without question the level of government responsible for acting in a national emergency under the peace, order and good government power (Monahan et al., 2017: 264–68), and if the minister is charged with CCG, then there is no reading of the Canadian Constitution that omits the principle of federalism or participation of the provinces going forward. For one, the provinces would be needed for a very familiar reason—passing necessary constitutional amendments. Under most situations that one could contemplate here, at least seven of the provinces with 50 per cent of the population (a number surely read with some flexibility under those circumstances) would be needed, and unanimous provincial consent would be needed for many major changes. The territories also pose their own problems. In a strict sense, they could probably be legislated away by the federal government should they choose, although the dual nature of Nunavut as a land claim poses its own problems from a constitutional perspective.

Indeed, this analysis also omits any reference to the inclusion of an Indigenous representative. The Constitution both explicitly and through interpretation requires consultation whenever Indigenous rights are being affected (Canada, 1982, ss. 35, 35.1), and while it would be impossible to provide for every Indigenous group, certainly some thought would have to go into ensuring that Indigenous interests are part of the picture. Thus, taken together, at a minimum, it is probably not possible to contemplate the continuation of constitutional government with anything less than about 1,000–1,500 people, at which point it might be possible to hold together a framework that would meet any definitions of the requirement. In addition, there are a number of smaller issues that would have to be taken into account. Cunningham notes that the seat of government in Canada is Ottawa, according to section 16 of the Constitution Act, 1867—something that would have to be changed in the event that a disaster made it uninhabitable (Cunningham, 2013: 90; Canada, 1867). The preservation of some objects, such as the Great Seal of Canada and the provincial equivalents, might be worth considering, although probably not strictly necessary.

## Conclusion

The current crisis is, of course, nowhere near the magnitude of an event requiring the implementation of plans based on the full CCG concept, and it is more limited to the realm of emergency management or possibly some aspects of continuity of government. But this is also an opportune moment to reflect on how the CCG concept might benefit from a more concrete definition, should it ever be required. The COVID-19 pandemic is probably the most significant global political and economic crisis since the Second World War. In the midst of these events, we are forced to seriously consider who really needs to go to work, what functions of the state really must continue, and what, on reflection, can be put off to another day.

## Notes

1 The literature on both COG in the United States, specifically, and on rights post-9/11, in general, is vast. No effort is made to canvass it here. For those interested in post-9/11 COG literature in the United States, a good start is the special edition of the *Catholic University Law Review* dedicated to the subject (see “Symposium on Ensuring the Continuity of Government in Times of Crisis,” 2004).

2 These tables have been edited from the original where updating was necessary and to list people otherwise included in the study's text but not in the tables, such as rough numbers needed for “additional administrative capacity.”

## References

[ref1] Canada. 1867. Constitution Act, 1867. 30 & 31 Victoria, c. 3 (UK) (Consolidated with Amendments).

[ref2] Canada. 1982. Constitution Act, 1982, being Schedule B to the Canada Act (UK) 1982.

[ref3] Canada. 2007. Emergency Management Act, S.C. 2007, c. 15.

[ref4] Cunningham, Lieutenant-Colonel J. W. 2013 “The Need for a Canadian Continuity of Government Policy: Being There When Canadians Need It Most.” Master's thesis. Canadian Forces College, Toronto, Ontario. https://www.cfc.forces.gc.ca/259/290/299/286/cunningham.pdf.

[ref5] Federal/Provincial/Territorial Ministers Responsible for Emergency Management. 2017 An Emergency Management Framework for Canada. 3rd ed. Ottawa: Emergency Management Policy and Outreach Directorate, Public Safety Canada https://www.publicsafety.gc.ca/cnt/rsrcs/pblctns/2017-mrgnc-mngmnt-frmwrk/2017-mrgnc-mngmnt-frmwrk-en.pdf.

[ref6] Graff, Garrett M. 2017 Raven Rock: The Story of the U.S. Government's Secret Plan to Save Itself. New York: Simon & Schuster.

[ref7] Monahan, Patrick, Byron Shaw and Padraic Ryan. 2017. Constitutional Law. 5th ed. Essentials of Canadian Law. Toronto: Irwin Law.

[ref8] Roach, Kent. 2008 “Ordinary Laws for Emergencies and Democratic Derogations of Rights” In Emergencies and the Limits of Legality, ed. Victor V. Ramraj. Cambridge: Cambridge University Press.

[ref9] “Symposium on Ensuring the Continuity of Government in Times of Crisis.” 2004 Catholic University Law Review 53 (4). https://scholarship.law.edu/lawreview/vol53/iss4/.

[ref10] Wilson, V. Seymour and Daniel Osabu-Kle. 1990 The Continuation of Constitutional Government in Canada: A Study Prepared for EPC. Ottawa: Emergency Preparedness Canada KE 4713 C66 1990. https://www.publicsafety.gc.ca/lbrr/archives/ke%204713%20c66%201990-eng.pdf.

